# Evaluation of newborn hearing screening program in Jordan

**DOI:** 10.3389/fped.2024.1420678

**Published:** 2024-07-11

**Authors:** Faten S. Obeidat, Noura Alothman, Rania Alkahtani, Sameer Al-Najjar, Mohammad Obeidat, Asia Y. Ali, Elham Ahmad, Alia A. Alghwiri

**Affiliations:** ^1^ Department of Hearing and Speech Sciences, School of Rehabilitation Sciences, University of Jordan, Amman, Jordan; ^2^ Department of Health Communication Sciences, College of Health and Rehabilitation Sciences, Princess Nourah bint Abdulrahman University, Riyadh, Saudi Arabia; ^3^ Department of Genetic & Congenital Disorders Prevention, Non-Communicable Diseases Directorate, Ministry of Health, Amman, Jordan; ^4^ Department of Audiology, Al-Bashir Hospital, Amman, Jordan; ^5^ Department of Information System and Program, Ministry of Health, Amman, Jordan; ^6^ Department of Physiotherapy, School of Rehabilitation Sciences, University of Jordan, Amman, Jordan; ^7^ Department of Physical Therapy, School of Health and Rehabilitation Sciences, Pittsburgh, PA, United States

**Keywords:** hearing screening, hearing loss, newborn, otoacoustic emission, auditory brainstem response, Jordan

## Abstract

**Introduction:**

The Newborn Hearing Screening (NHS) program was officially launched in Jordan in 2021. Since its inception, no studies have examined the effectiveness of the program. This study seeks to assess the effectiveness and outcomes of the NHS program in Jordan.

**Methods:**

A retrospective cross-sectional study was conducted to investigate the program coverage rate, referral rate, loss to follow-up rate and the hearing status of newborns who successfully completed the necessary diagnostic assessment. Live births in all hospitals administered by the Ministry of Health (MoH) in Jordan from July 2021 to November 2023 were included.

**Results:**

Out of 25,825 newborns delivered, 99.4% (25,682) were screened. A referral rate of 0.7% (189) was recorded. Approximately 61.9% of those referred (*n* = 117) had normal hearing, while 31.7% (60 infants) were diagnosed with hearing loss. The prevalence of congenital hearing loss was 0.14%, and the mean age for identifying hearing loss was 11 months.

**Discussion:**

The current status of the NHS program in Jordan is promising. The program has achieved most benchmarks recommended by the Joint Committee on Infant Hearing (JCIH), demonstrating encouraging outcomes. There is a need to investigate and address the factors causing delays in the identification of hearing loss in Jordan.

## 1 Introduction

Newborn hearing screening (NHS) programs have become an integral component of early childhood healthcare across the globe. The aim of these programs is to identify permanent hearing impairments in infants as early as possible, allowing for timely intervention and support, while minimizing false positives to prevent unnecessary expenses and alleviate parental concerns (1). The success of a screening program is closely tied to the efficiency of the diagnostic follow-up process. Disparities in healthcare systems worldwide can influence the speed and accessibility of follow-up assessments, impacting the overall success of NHS initiatives. Additionally, cultural attitudes and practices regarding healthcare, disability, and hearing impairment influence the success of screening programs and the willingness of parents to engage in follow-up and intervention services (2).

Several studies conducted internationally have assessed the effectiveness of NHS programs by employing various quality metrics, including coverage rate, referral rate and follow-up rate. The coverage rate, indicative of the accessibility and acceptance of the NHS, is targeted to exceed 95%–97%. Referral rate, denoting the proportion of infants identified with screening failure and subsequently referred for diagnostic assessment, typically stands at 4% for all infants (3–6). The follow-up rate, defined as the percentage of infants whose caregivers attend the follow-up appointment subsequent to screening failure or referral for diagnostic assessment, should not fall below 90% (3).

In 2021, the World Health Organization (WHO) published the first World Report on Hearing, where the significant role of the NHS in integrated people-centered ear and hearing care (IPC-EHC) was acknowledged. It also highlighted “ensuring widespread coverage of newborn hearing screening services among the population” as one of three key indicators for globally monitoring and assessing advancements in ear and hearing care. As part of efforts to augment IPC-EHC, the WHO advocated for a 20% augmentation in the coverage rate of NHS programs by the year 2030. Specifically, nations with a coverage rate of less than 50% were advised to elevate it to a minimum of 50%, those with a coverage rate ranging from 50%–80% were recommended to implement a 20% increase, and nations with a coverage rate equal to or exceeding 80% were advised to achieve universal coverage.

The outcomes of NHS programs often vary based on factors such as the screening methods employed, healthcare infrastructure, and cultural considerations. Mackey et al. (7) conducted an evaluation of the NHS programs in 47 countries, revealing a coverage rate ranging from 97%-100% and a referral rate of less than 4% for low-risk infants and equal to or greater than 4% for high-risk infants. Regarding the follow-up rate, significant variability was observed in Mackey et al.'s (7) findings, with rates ranging from 27%–97% after referral for a second screening step and 19%–97% after referral for diagnostic assessment. Consistent with these observations, Neumann et al. (2) reported persistent global disparities in their study, which incorporated data from 158 countries. Their findings indicated that less than one third of infants worldwide participate in screening programs with a coverage rate of equal to or greater than 85%. Neumann et al. (2) also noted an average of 4.5% of infants failing the screening test, leading to referral for diagnostic assessment, with a subsequent 17.2% loss-of-follow-up rate. Disparities were evident between high-income countries on one side and low- and middle-income countries on the other.

In 2021, the NHS program was initiated by the Ministry of Health (MoH) in Jordan. This program includes all hospitals within the MoH sector, comprising 23 hospitals with maternity wards, and its goal to screen every newborn within 72 h after birth or before discharge. The protocol mandates the utilization of Transient Otoacoustic Emission (TEOAE) for the initial hearing screening of each newborn. If a newborn fails this screening, the established procedure involves referring the infant to the audiologic clinic for a second-step hearing screening and, if necessary, diagnostic assessment if suspected with unilateral or bilateral hearing loss.

To the best of the author's knowledge, none of the previous studies have examined the effectiveness of the national NHS program since its launch in 2021. Therefore, the aim of the present study was to evaluate the NHS program's effectiveness and outcomes in Jordan. This evaluation provides valuable insights into the efficacy of the implemented measures and aids in refining the screening program for enhanced effectiveness and positive outcomes for the targeted population.

## 2 Methods

The study received approval from the Institutional Review Board (IRB) under the registration number MOH/REC/2023/382.

### 2.1 Study population

A retrospective descriptive cross-sectional study was conducted on newborns screened for hearing loss between July 2021 and November 2023 across all hospitals within the MoH sector. A total of 23 birthing hospitals were included in the study. NHS data were obtained from the MoH, while diagnostic results for newborns referred from screening were collected by a third party after obtaining a permission. The evaluation focused on the number of newborns screened, pass rates, referral rates, and rates of loss to follow-up to gauge the effectiveness of the NHS program in Jordan. For those who referred for diagnostic assessment, the results of the assessment were collected (normal hearing vs. hearing loss). The age of identification of hearing loss was also recorded.

### 2.2 Protocol

The hearing screening was obtained using TEOAE. The screening for healthy newborns took place within the first 72 h after delivery or, if applicable, before discharge-whichever occurred first. For neonates in the Neonatal Intensive Care Unit (NICU) the screening is conducted just before discharge. An audiologist or trained nurse supervised by an audiologist administered the screening. The testing was conducted in a nursery quiet room, which, while not perfectly soundproofed, is situated away from noise. All acoustic sources were removed to create a conducive environment for the testing process.

Two stages of the screening were implemented for newborns. During the first stage, newborns were screened before discharge, with separate screening for each ear. Results were categorized as either “Pass” or “Refer.” Newborns with “Refer” results in one or both ears were scheduled for a second screening in the audiology department 2-3 weeks post-delivery. If one or both of the newborn's ears fail the second screening test, the patient is referred to the audiology clinic for the diagnostic Auditory Brainstem Response (ABR) test, which is conducted by an audiologist between 3 and 6 months of age.

Screenings were conducted using the Sentiero by PATH medical GmbH equipment, a German manufacturer. A click ranging from 1 to 4 KHz at 75 dB SPL was used. A “Pass,” indicating normal hearing, was determined with a signal-to-noise ratio of of a minimum of 6 dB, evident in at least three frequencies.

## 3 Results

In total, 25,825 newborns were delivered during the period from July 2021 to November 2023. A total of 25,682 newborns were screened for their hearing with a coverage rate of 99.4%. Due to a technical error, the pass and referral rates of the initial screening stage were not recorded in the MoH records, and therefore only the results of the second screening stage and the diagnostic stage were reported here.

**Figure 1:**
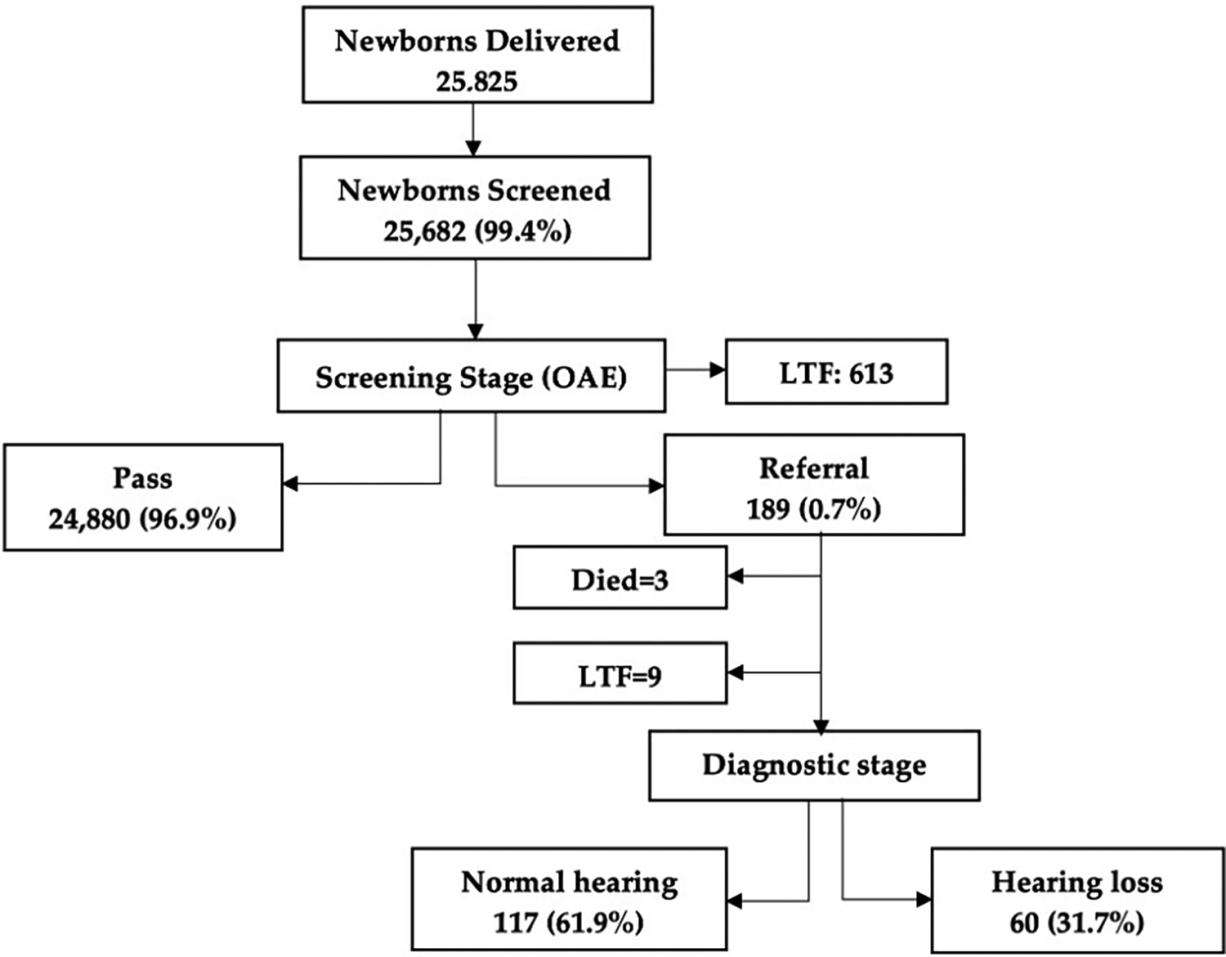
Flowchart of screening and diagnostic stages of the newborn hearing screening program. LTF, loss to follow-up.

During the screening stage, the pass rate was 96.9% (*n* = 24,880). The referral rate for one or both ears was found to be 0.7% (*n* = 189; 86 male and 103 female; 72 with a risk factor for hearing loss). All the 189 newborns who failed the screening were referred for diagnostic ABR. Of them, 31.7% were diagnosed and confirmed to have hearing loss (*n* = 60), whereas 61.9% were confirmed to have normal hearing (*n* = 117). It should be noted that 613 newborns did not show up for their second screening stage, resulting in a loss to follow-up (LTF) rate of 2.4%. Additionally, during the diagnostic stage, three newborns were deceased before their diagnostic appointments (1.6%), and nine newborns did not show up for their diagnostic appointments (LTF = 4.8%) (Figure 1).

Table 1 shows the types of hearing loss for those diagnosed with hearing loss. Out of the 60 newborns who were diagnosed with hearing loss, 55% of them were diagnosed with sensorineural hearing loss bilaterally (*n* = 33), 40% had conductive hearing loss either bilaterally (*n* = 15) or unilaterally (*n* = 9), and 3.3% had mixed hearing loss either bilaterally (*n* = 1) or unilaterally (*n* = 1). The mean age of identification of hearing loss was 11 months (standard deviation: ± 6.3 months).

**Table 1:** Types of hearing loss for the 60 newborns diagnosed with hearing loss.

Type of hearing loss		*n* (%)
Sensorineural	Bilateral	33 (55%)
	Unilateral	0 (0%)
Conductive	Bilateral	15 (25%)
	Unilateral	9 (15%)
Mixed	Bilateral	1 (1.6%)
	Unilateral	1 (1.6%)
Unknown	Bilateral	1 (1.6%)
	Unilateral	(0%)

## 4 Discussion

The NHS has been globally adopted to identify all newborns who are at risk of hearing loss as early as possible (1). Globally, untreated hearing loss ranks as the third most significant contributor to years lived with disability (8). Early hearing detection and intervention activities have significantly benefited children who are deaf or hard of hearing and their families (1). Starting with a hearing test shortly after birth is crucial for early identification of hearing problems.

In 2021, Jordan implemented the NHS program in public hospitals, aiming to identify hearing concerns shortly after birth, allowing for timely intervention and support. This study is the first to present a comprehensive overview of the program nationally in Jordan. This study primarily focuses on the program's coverage rate, as well as the pass and referral rates, providing an initial overview of its performance at the national level.

### 4.1 Coverage rate

Adequate screening coverage is vital for NHS programs to confirm that all newborns undergo screening without any being missed. The NHS program coverage rate in Jordan was found to be 99.4%, aligning well with the JCIH recommendation of over 95% (3, 9) and surpassing many countries worldwide. Comparatively, data from the United States, including the 2019 CDC summary and Early Hearing Detection and Intervention (EHDI) data, indicated a slightly lower coverage rate of 98.4% (10). Notably, the U.S. NHS program, a decade into its implementation, had a considerably lower coverage rate of 70% for newborns screened before hospital discharge in 2002 (11). In Saudi Arabia, a 2024 report revealed a coverage rate of 92.6% (12), while Oman estimated a coverage rate of approximately 90% by 2020 (13). Europe reported an 80% coverage rate in 2010, encompassing 80% of European countries that had implemented nationwide NHS programs (14).

### 4.2 Referral and loss to follow-up rates

The study indicates an overall referral/fail rate of 0.7% across all MoH hospitals in Jordan following the initial two-stage screening, consistent with the JCIH recommendation of less than 4% (3). The referral rate in the current study is comparable to the approximately 0.7% reported in Saudi Arabia after two-stage screening protocol (15). Another recent study by Alothman et al. reported a higher referral rate of 1.87% across all provinces of Saudi Arabia following a three-stage screening protocol (16). In Oman, the NHS program indicated a referral rate of 6.6%, surpassing the internationally recommended maximum benchmark of 4% (13).

The reported loss to follow-up rate in the current study was 2.4% for the screening stage and 4.8% for the diagnostic stage, falling below the JCIH recommendation of 10% or less (3). This rate was significantly lower than that reported in a tertiary hospital in Saudi Arabia, where an 18% loss to follow-up rate was documented (12).

### 4.3 Prevalence of hearing loss

The epidemiological data available on hearing loss in Jordan is limited. In 2014, Abu-Shaheen et al. (17) estimated the occurrence of congenital hearing loss in 1.5% of live births in Jordan. A more recent study reported a prevalence of 1% among infants admitted to NICU and 0.07% among infants in well-baby nurseries (18), surpassing international data. For instance, a systematic review and meta-analysis study reported that the prevalence of permanent hearing loss worldwide ranged from 1 to 6/1,000 with an overall prevalence of 2.2/1,000 (19).

In our study, the occurrence of hearing loss in infants was 0.14%, consistent with the global prevalence of neonatal hearing impairment, which falls within the range of 0.1%–0.3% (20). This figure, however, exceeded the findings of the Nuseir study, 0.07% (18). It's worth noting that the Nuseir study involved only one hospital in the north of Jordan whereas our study involved all MoH hospitals.

### 4.4 Age of hearing loss identification

The mean age for identifying hearing loss in our study was 11 months (standard deviation: ± 6.3 months). This is higher than the international recommendation of 3 months old (3). In the USA, the average age of identification is around 3 months (21), in the UK an average of 49 days is reported (22). On the other hand, our findings surpass those reported in other Middle East countries such as in Saudi Arabia where an average of 3 years old was reported (23). However, it is worth mentioning that the finding from the KSA was reported prior to the implementation of the NHS in the country.

It is crucial to investigate and address the reasons behind the delayed age of identifiying hearing loss in children in Jordan, particularly given the nationwide implementation of the NHS program with a coverage rate of 99.4%. These reasons may include limited access to audiology clinics, long waiting lists and delays in scheduling diagnostic appointments, caregiver scheduling preferences that result in delays such as specific time constraints, requiring extensive diagnostic testing and lack of resources and personnel.

Addressing these challenges may involve improving healthcare infrastructure, streamlining referral processes, increasing the availability of qualified professionals, and implementing strategies to reduce waiting times for diagnostic assessments. It is imperative to earnestly address the task of minimizing the age at which hearing loss is detected in children. Failure to do so may engender adverse consequences, detrimentally impacting the child's linguistic acquisition, speech perceptibility, socio-emotional well-being, and scholastic performance, as elucidated by authoritative sources such as the WHO (24) and the American Academy of Pediatrics (25). Moreover, this delay in identification and subsequent intervention has far-reaching implications on the affected individuals' prospective employability and work-related productivity, thereby incurring an additional economic burden on society (26). These repercussions could have otherwise been mitigated or, at the very least, ameliorated through timely preventive measures.

### 4.5 Recommendations

Enhancing the existing national NHS program in Jordan should involve incorporating automated ABR into the screening protocol. A limitation of the NHS program in Jordan was its exclusive reliance on TEOAE testing for screening. Diagnostic ABR was only conducted for infants who did not pass the OAE testing. Attias et al. conducted a study revealing a relatively elevated prevalence of auditory neuropathy (1.37%) among Jordanian newborns who passed the OAE testing, especially when contrasted with a non-Jordanian population (27). It's important to note that OAE testing cannot detect auditory neuropathy (28).

In Jordan, newborns who fail the hospital screening are directed for follow-up audiological assessment, with the diagnostic ABR intended to be conducted between 3 and 6 months. However, it is emphasized that adhering to the JCIH recommendation is vital, indicating that every newborn referred from the NHS should undergo diagnostic ABR before 3 months of age (3). It is crucial to complete the diagnostic assessment for infants referred from the NHS before 3 months of age, in alignment with JCIH guidelines. This can effectively address the risk of delayed identification of hearing loss in Jordan.

The current dataset does not include infants in private and non-MoH sector hospitals. It is essential to integrate all non-governmental hospitals into the national NHS program for comprehensive coverage and effectiveness.

## 5 Conclusion

As per the current study, the NHS program demonstrated a coverage rate of 99.4%, a referral rate of 0.7%, and a 0.14% prevalence of hearing loss among infants. The mean age for identifying hearing loss was 11 months with a standard deviation of ±6.3 months.

The data retrieved in this study, despite reflecting the program's performance within MoH hospitals in Jordan, indicate promising results in coverage and referral rates. Notably, these performance measures, observed two and a half years after the program's inception, are comparable to successful international standards. It is essential to note that the reported data solely pertain to MoH hospitals, as non-MoH and private hospitals are presently not obligated by the government to adopt the UNHS program.

## Data Availability

The datasets presented in this article are not readily available due to data confidentiality. Requests to access the datasets should be directed to faten.oebidat@ju.edu.jo.
